# Study on Electrochemical Insulin Sensing Utilizing a DNA Aptamer-Immobilized Gold Electrode

**DOI:** 10.3390/ma8084710

**Published:** 2015-07-24

**Authors:** Izumi Kubo, Taiga Eguchi

**Affiliations:** Graduate School of Engineering, Soka University, 1-236 Tangi, Hachioji, Tokyo, 192-8577, Japan; E-Mail: 1s.gt-t.feel-coolest@ezweb.ne.jp

**Keywords:** DNA aptamer, hemin, insulin, glucagon, G-quadruplex, peroxidase activity, gold electrode

## Abstract

We investigated an insulin-sensing method by utilizing an insulin-binding aptamer IGA3, which forms an anti-parallel G-quadruplex with folded single strands. Spectroscopic observation indicates that some anti-parallel G-quadruplex bind hemin and show peroxidase activity. In this study, the peroxidase activity of IGA3 with hemin was confirmed by spectrophotometric measurements, *i.e.*, the activity was three-times higher than hemin itself. IGA3 was then immobilized onto a gold electrode to determine its electrochemical activity. The peroxidase activity of the immobilized IGA3-hemin complex was determined by cyclic voltammetry, and a cathodic peak current of the electrode showed a dependence on the concentration of H_2_O_2_. The cathodic peak current of the IGA3-hemin complex decreased by binding it to insulin, and this decrease depended on the concentration of insulin.

## 1. Introduction

Enzymes, receptors and antibodies are utilized as recognition elements of biosensors. These molecules recognize their target selectively, but they are proteins and are thus labile to heat or acidic conditions. In biological systems, it is known that certain nucleic acid sequences, such as DNA and RNA molecules, bind to a particular molecule, such as transcription factors or regulatory molecules. In other words, some DNA or RNA sequences recognize target molecules selectively. Nucleic acid (or RNA) sequences that have specific binding ability are termed DNA (RNA) aptamers. Many aptameric sequences are registered in libraries, and aptamers to various target molecules are available [1,2,3]. Generally speaking, DNA is more stable than RNA, which is easily degraded by RNase in saliva and sweat. DNA can withstand heat and extreme conditions more than protein. Consequently, DNA aptamers can act as stable recognition elements of biosensors. As a result, their application to biosensors has been increasingly investigated [4,5,6,7]. 

On the other hand, DNA oligomers form their own tertiary structure depending on their sequence. There are several types of tertiary structures in DNA molecules. The most well-known double-helical structures are A-DNA, B-DNA, C-DNA and Z-DNA [8,9,10]. Single-stranded DNA sequences often form a hairpin structure through hydrogen bonding between the adenine-thymine or the cytosine-guanine base pair [11]. Although these base pairs are most frequently observed in the structure of nucleic acid sequences, some guanine-rich DNA oligomers form a G-quartet comprised of four hydrogen-bonded guanine bases associated via Hoogstein base-pairing [12]. In the structure of a G-quartet, four guanine bases form a plane, and when G-quartet planes are stacked, this is called a G-quadruplex [13]. A G-quadruplex that encloses hemin constitutes the complex of protoporphyrin with ferric ion in the center, and this binding enhances the degradation activity of hydrogen by hemin.

In 1998, Travascio and co-workers reported that a DNA aptamer-hemin complex exerted peroxidase activity following spectrophotometric measurements [14]. They demonstrated the peroxidase activity of the hemin-binding G-quadruplex, named PS2M, using two substrates, hydrogen peroxide (H_2_O_2_) and an electron-donating compound, 2,2′-azino-bis(3-ethylbenzothiazoline-6)-sulfonic acid (ABTS), which is colorless. When H_2_O_2_ is reduced, oxidized ABTS, which is green, increases, and peroxidase activity can then be observed by visible light spectroscopy. Although spectroscopic measurements to measure activity are easy and commonly used, sufficient molarity of H_2_O_2_ (0.75 mM) is necessary to cause a detectable change in the absorption change of ABTS. Thus, kinetic studies to determine the Michaelis constant are difficult when using spectrophotometry.

Instead of using ABTS, we demonstrated in our previous study [15] that the electrochemical nature of G-quadruplex peroxidase activity could be measured by modifying a gold electrode with a DNA aptamer, such as PS2M. In our method, electrons were donated by the gold electrode to H_2_O_2_ through hemin. Without ABTS, the change in cathodic peak current of the hemin-binding G-quadruplex was measured, and the Michaelis constant of PS2M was determined. This method offers not only a kinetic measurement, but also the potential to apply the DNA aptamer-based modified electrode to biosensors.

Yoshida *et al*. [16] selected, as a DNA aptamer to insulin, IGA3 (5′-GGTGGTGGGGGGGGTTGGTAGGGTGTCTTC-3′) by the SELEX (Systematic Evolution of Ligands by Exponential enrichment) method. This guanine-rich aptamer forms an anti-parallel G-quadruplex that binds insulin selectively. However, whether it shows peroxidase activity by aiding hemin or not has not yet been investigated. 

**Figure 1:**
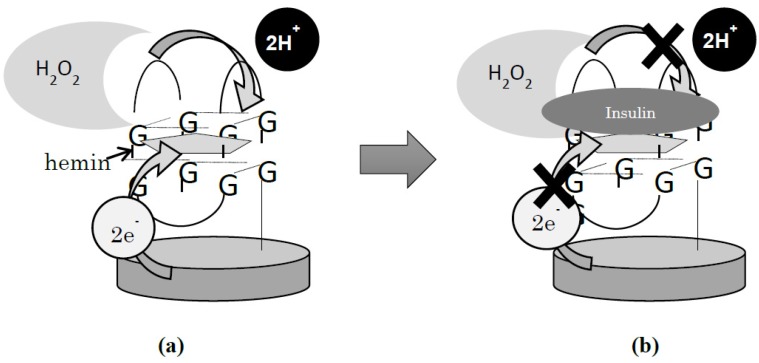
Scheme of insulin sensing utilizing a G-quadruplex immobilized electrode: (**a**) measurement of peroxidase activity with the aid of hemin without insulin; (**b**) measurement of peroxidase activity after binding to insulin.

We assumed that IGA3 would indicate peroxidase activity with the aid of hemin. If IGA3 with hemin displays peroxidase activity, it can be detected electrochemically, as well as with PS2M (Figure 1a). It is predicted that the peroxidase activity of IGA3 will be affected by binding it to insulin. Since insulin will block the access of H_2_O_2_ to hemin, such interference should be determined by an electrochemical measurement (Figure 1b). The objective of this study was to establish a method to electrochemically sense insulin based on its interference of peroxidase activity by IGA3, the DNA aptamer. Selectivity of IGA3 to insulin was examined by comparing with the response to glucagon, which is a polypeptide hormone like insulin. 

## 2. Results and Discussion

### 2.1. Spectroscopic Activity of IGA3

**Figure 2:**
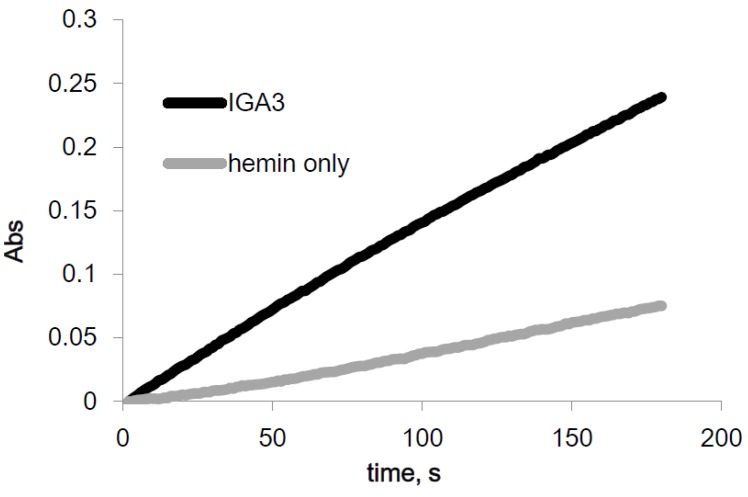
Time course in absorbance of oxidized 2,2′-azino-bis(3-ethylbenzothiazoline-6)-sulfonic acid (ABTS) by hemin and the IGA3-hemin complex.

The peroxidase activity of the IGA3-hemin complex was determined and compared to that of hemin by a spectrophotometric method using ABTS as the electron donor. The initial absorbance change at 414 nm, which is the λ_max_ of oxidized ABTS, was measured for 180 s. The time course of the absorbance is shown in Figure 2. The absorbance of both hemin and the IGA3-hemin complex increased throughout the measurement. The increase in absorbance of the IGA3-hemin complex was more than three-times larger than that of hemin. Thus, IGA3 enhanced the peroxidase activity of hemin by forming a complex. 

**Figure 3:**
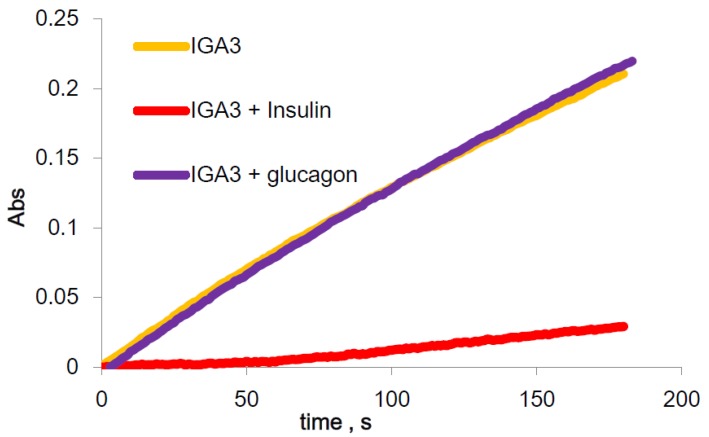
Peroxidase activity of the IGA3-hemin complex and the activity change by insulin and glucagon.

The peroxidase activity of IGA3 with or without insulin was then examined after incubation with 10 μM insulin by spectroscopic time course measurements, as shown in Figure 3. 

The increase in absorbance after 180 s with insulin was 0.02 ± 0.006, but without insulin, it was 0.24 ± 0.01. Oxidation of ABTS by the IGA3-hemin complex was considerably reduced after binding with insulin. This implies that insulin interferes with the peroxidase activity of IGA3. To confirm the specificity of IGA3, the change in absorbance after incubation with 10 μM glucagon was examined (Figure 3), because glucagon is a peptide hormone as insulin and regulates glucose in blood; its molecular weight is 3.5 kD, which is not so different from the molecular weight of insulin, 6.5 kD. The change in absorption was 0.26 ± 0.02 and was almost the same as for IGA3 without binding to insulin. This indicates that insulin binds to IGA3 and affects its peroxidase activity, while glucagon does not bind to IGA3 and does not change its peroxidase activity. These facts indicate that IGA3 binds to insulin selectively. Although Yoshida *et al*. reported the G-quadruplex formation of IGA3 and its binding to insulin [16], its selectivity to insulin compared to glucagon was not studied. 

### 2.2. Electrochemical Response of the IGA3-Hemin Complex

#### 2.2.1. Peroxidase Activity of the Immobilized IGA3-Hemin Complex

**Figure 4:**
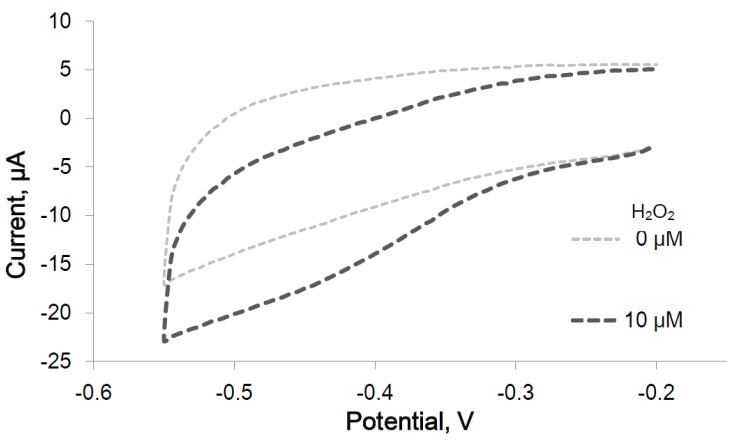
Cyclic voltammogram of the IGA3-hemin complex with and without hydrogen peroxide; the scan rate is 10 V s^−1^.

After peroxidase activity of the IGA3-hemin complex was confirmed, it was examined electrochemically using an IGA3-modified electrode. After the immobilization of IGA3 and the formation of the G-quadruplex on a gold electrode surface, peroxidase activity of the IGA3-hemin complex was examined by cyclic voltammetry (CV). Figure 4 shows the typical CV of the IGA3-hemin complex. Without H_2_O_2_, no cathodic current change was observed. After the addition of 10 μM H_2_O_2_, the cathodic current decreased below −0.3 V. The difference in current with or without H_2_O_2_ was evaluated from a cyclic voltammogram, and a cathodic peak was observed at −0.45 V with various concentration of H_2_O_2_. 

**Figure 5:**
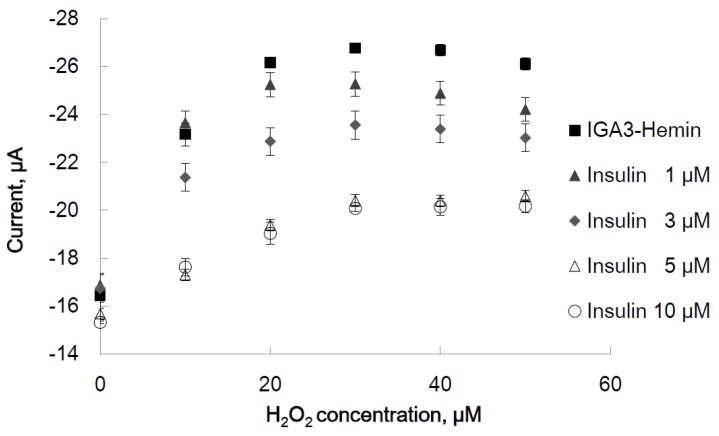
Peroxidase activity of the IGA3-hemin complex and the effect of insulin on its cathodic peak current activity at −0.45 V was plotted *vs.* H_2_O_2_ concentration, *n* = 3.

At various concentrations of H_2_O_2_ (10, 20, 30, 40 and 50 μM), CV was measured, and a cathodic peak current of each concentration was obtained at the same potential. The peak current was plotted *versus* the concentration of H_2_O_2_, as shown in Figure 5 (IGA3-hemin). The absolute value of the cathodic peak current increased as the concentration of H_2_O_2_ increased to 30 μM, and at higher concentrations, it reached a steady state. It was demonstrated that the IGA3 hemin-complex, as well as PS2M have peroxidase activity. To determine the Michaelis constant of the enzyme, reaction velocity should be plotted for the concentration of substrate at a certain concentration of the enzyme as in Figure 5. From the curve fit to the plots, maximum reaction velocity (*V*) can be obtained, and the Michaelis constant is defined as the substrate concentration at V/2 of the curve. We assume IGA3 works as peroxidase, and the reaction velocity is determined as the cathodic peak current at each concentration of H_2_O_2_. In Figure 5, the reaction velocity of IGA3 was plotted for substrate H_2_O_2_. From the plots of IGA3-hemin, *V* and the Michaelis constant of the IGA3-hemin complex was obtained. We evaluate the Michaelis constant of the IGA3-hemin complex from the averaged current, because the errors of the plots are as small as 5%. The Michaelis constant of the IGA3-hemin complex was determined as 8.0 μM of H_2_O_2_ from Figure 5. We previously reported the Michaelis constant of PS2M to be 23 μM [15]. The Michaelis constant of IGA3 was smaller than that of PS2M, and the activity of IGA3 is higher than that of PS2M. It was considered that the difference in the Michaelis constant might be caused by the difference in the distance of hemin from the surface of the electrode, because it was observed that the hemin, located at a longer distance from the electrode surface, resulted in a greater Michaelis constant in our previous study [15]. Hemin in IGA3 might be located nearer than that in PS2M.

#### 2.2.2. Effect of Insulin on Peroxidase Activity of the IGA3-Hemin Complex

After observing the interference of peroxidase activity of the IGA3-hemin complex by insulin, this interference was measured electrochemically. To investigate the effect of insulin on the peroxidase activity of the IGA3-hemin complex, after the formation of the G-quadruplex, the IGA3-immobilized electrode was immersed in various concentrations of insulin (1, 3, 5 and 10 μM) for 30 min at room temperature and rinsed with water. Then, peroxidase activity was determined. 

**Figure 6:**
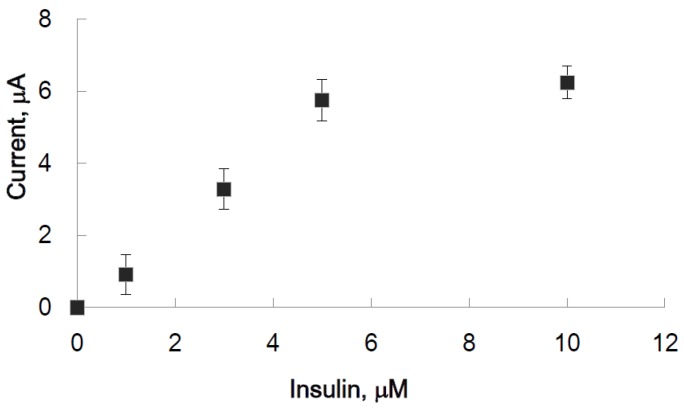
Calibration plot of current difference to insulin concentration. The cathodic peak current difference between the state with and without insulin was plotted *versus* insulin concentration. The concentration of H_2_O_2_ was 20 μM.

By immersing in insulin solution, insulin would bind to IGA3, and the diffusion of H_2_O_2_ to hemin would be disturbed and the absolute value of the cathodic peak current decreased. As shown in Figure 5, without H_2_O_2_ at every concentration of insulin, almost the same peak current was observed. However, after immersion in insulin solution, the current value decreased, and a simultaneous decrease in peroxidase activity was observed. The decrease in peroxidase activity depended on the concentration of insulin at every concentration of H_2_O_2_. The effect to cathodic peak current was obviously observed at 20 μM H_2_O_2_. The difference between cathodic peak current with and without insulin was obtained as the difference in cathodic peak of each concentration of insulin. The difference in cathodic peak current, when the concentration of H_2_O_2_ was 20 μM, was plotted (Figure 6). A linear relationship between the concentration of insulin and cathodic peak difference was observed in the range of 0–5 μM insulin.

#### 2.2.3. Specific Binding to Insulin

**Figure 7:**
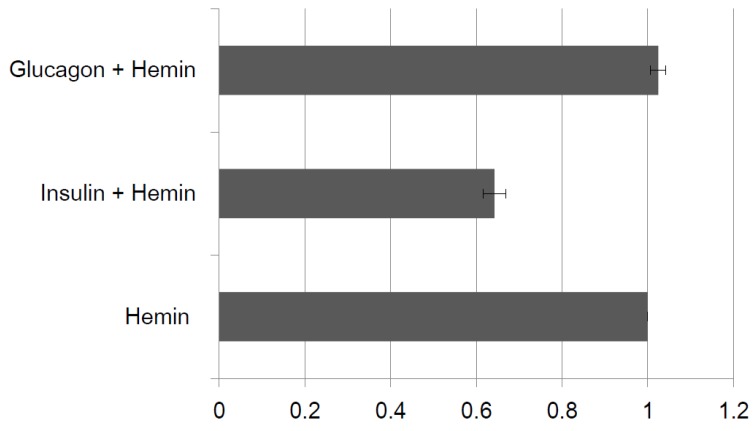
Selectivity of the IGA3 hemin-immobilized electrode. The ratio of cathodic peak current of each condition to that of the IGA3-hemin immobilized electrode (hemin) is shown.

Selectivity was confirmed by electrochemical measurements as in spectrophotometric measurements. After IGA3 was immobilized to a gold electrode, selectivity was examined using glucagon. When H_2_O_2_ was 20 μM, the cathodic peak current of the IGA3-hemin complex was compared to that after incubation with insulin (3 μM) and glucagon (3 μM). Although the cathodic peak current after immersion of the IGA3-modified electrode with insulin decreased to 62%, it did not change when immersed in glucagon solution, unlike insulin (Figure 7). This indicates that glucagon does not bind to IGA3. This result is consistent with the spectrophotometric observation. Therefore, the IGA3-hemin complex binds insulin selectively.

## 3. Experimental Section

### 3.1. Materials

Hemin (ferriprotoporphyrin IX chloride) was purchased from Sigma Aldrich (Tokyo, Japan) and used without further purification. ABTS (biochemistry grade) was purchased from WAKO Pure Chemicals (Osaka, Japan). Other reagents were of laboratory grade and were used without any purification.

IGA3, the DNA aptamer used in this study, was synthesized and supplied by Operon Biotechnology (Tokyo, Japan). At the 5′ end of the DNA, a thiol group was added for immobilization to the electrode, and to help with the formation of the G-quadruplex, a poly A linker (4A) was inserted between thiol and the aptamer sequence according to our previous study [15]. Electrochemical reactions in this study were carried out in 40 K buffer solution of pH 6.5 (50 mM 2-morphorinoethanesulfonic acid (MES), 100 mM Tris acetate, 40 mM potassium acetate and 50 mM potassium chloride). Human recombinant insulin (JEN-PR477, Funakoshi, Tokyo, Japan) was used.

### 3.2. Spectrophotometric Measurement of Peroxidase Activity

The peroxidase activity of IGA3 with hemin was measured spectrophotometrically according to a previously-reported method [14]. IGA3 solution (6 μM, solubilized in 40K buffer was heated at 90 °C for 10 min to break the intramolecular hydrogen bonds, then cooled to room temperature and left for 1 h to form the G-quadruplex. To this solution, 5 μM hemin was added, mixed well, then incubated for 30 min to form the IGA3-hemin complex. ABTS solution and H_2_O_2_ were then added. The initial concentration of IGA3, hemin, ABTS and was 2.0 μM, 0.3125 μM, 2.5 mM and 0.75 mM, respectively. Immediately after the addition of H_2_O_2_, a time course of the absorbance at 414 nm, which is the peak wavelength of oxidized ABTS, was measured for 180 s using a UV/VIS spectrophotometer (UV2450, Shimadzu, Kyoto, Japan). 

### 3.3. Preparation of Hemin-Binding DNA Aptamer-Modified Electrode

A gold electrode (AUE, Φ 1.6mm, BAS) was used to immobilize IGA3. The DNA aptamer (IGA3) was immobilized onto the gold electrode as follows. The electrode was polished using aluminum slurry (0.05 μM) prior to immobilization. The polished electrode was sputtered by ion plasma in an ion coater (Eiko Engineering, Tokyo, Japan) for 5 min to obtain a completely cleaned surface. Immediately after plasma cleaning, the electrode was immersed in DNA solution (3 μM) and kept at 4 °C overnight. After immersion, the DNA-modified electrode was rinsed with pure water, and heat-induced denaturation of immobilized DNA oligomers (90 °C, 10 min) was carried out and followed by annealing in buffer containing K^+^ (40K buffer) at room temperature to allow the formation of the G-quadruplex. Then, the DNA-immobilized electrode was immersed in hemin solution (5 μM) for 10 min before electrochemical measurements. Thereafter, the hemin-binding DNA aptamer-modified electrode was prepared. 

### 3.4. Electrochemical Measurements

Electrochemical measurements were performed using a voltammetry analyzer (ALS1200, BAS, Tokyo, Japan) and a three-electrode electrochemical cell. The DNA-immobilized gold electrode served as the working electrode. An Ag/AgCl electrode and a platinum wire electrode were used as the reference and counter electrode, respectively. All electrochemical measurements were carried out in a glass vial containing 10 mL of deoxygenated 40K buffer solution at room temperature. Cyclic voltammetry was carried out to measure the peroxidase activity of the immobilized DNA and hemin complex according to our previously-reported procedure [15]. Every measurement was performed three times.

## 4. Conclusions

Peroxidase activity of a DNA aptamer (IGA3) hemin complex was confirmed by a spectrophotometric method using ABTS as the electron donor. Insulin, but not glucagon, binding was interfered with peroxidase activity. Peroxidase activity was determined not only by a spectroscopic method, but also by an electrochemical measurement using an IGA3-immobilized gold electrode without using an electron donor molecule, ABTS. While measuring cyclic voltammetry, cathodic peak current caused by the reduction of H_2_O_2_ was observed at −0.45 V, and a decrease in peak current was observed by insulin binding. Moreover, a linear relationship between cathodic peak difference and insulin concentration was observed at an insulin concentration range of 0–5 μM. The electrochemical response of IGA3 to insulin was selective. The IGA3-immobilized gold electrode can serve as an insulin sensor. This is the first report of the application of the detection of peroxidase activity of G-quadruplex DNA aptamer to a biosensor. 
